# Biomarkers and the Development of a Personalized Medicine Approach in Spinal Muscular Atrophy

**DOI:** 10.3389/fneur.2019.00898

**Published:** 2019-08-19

**Authors:** Didu S. T. Kariyawasam, Arlene D'Silva, Cindy Lin, Monique M. Ryan, Michelle A. Farrar

**Affiliations:** ^1^Department of Neurology, Sydney Children's Hospital, Sydney, NSW, Australia; ^2^School of Women's and Children's Health, University of New South Wales Medicine, University of New South Wales, Sydney, NSW, Australia; ^3^Department of Neurophysiology, Brain and Mind Center, University of Sydney, Sydney, NSW, Australia; ^4^Department of Neurology, Murdoch Children's Research Institute, Royal Children's Hospital, University of Melbourne, Melbourne, VIC, Australia

**Keywords:** biomarker, spinal muscular atrophy, motor unit number estimation, compound muscle action potential, *SMN2*, neurofilament

## Abstract

Recent unprecedented advances in treatment for spinal muscular atrophy (SMA) enabled patients to access the first approved disease modifying therapy for the condition. There are however many uncertainties, regarding timing of treatment initiation, response to intervention, treatment effects and long-term outcomes, which are complicated by the evolving phenotypes seen in the post-treatment era for patients with SMA. Biomarkers of disease, with diagnostic, prognostic, predictive, and pharmacodynamic value are thus urgently required, to facilitate a wider understanding in this dynamic landscape. A spectrum of these candidate biomarkers, will be evaluated in this review, including genetic, epigenetic, proteomic, electrophysiological, and imaging measures. Of these, *SMN2* appears to be the most significant modifier of phenotype to date, and its use in prognostication shows considerable clinical utility. Longitudinal studies in patients with SMA highlight an emerging role of circulatory markers such as neurofilament, in tracking disease progression and response to treatment. Furthermore, neurophysiological biomarkers such as CMAP and MUNE values show considerable promise in the real word setting, in following the dynamic response and output of the motor unit to therapeutic intervention. The specific value for these possible biomarkers across diagnosis, prognosis, prediction of treatment response, efficacy, and safety will be central to guide future patient-targeted treatments, the design of clinical trials, and understanding of the pathophysiological mechanisms of disease and intervention.

## Introduction

Spinal muscular atrophy (SMA) is characterized by progressive loss of motor neurons in the brainstem and spinal cord resulting in muscle weakness (1). It is the leading inherited cause of infant mortality with severity ranging from progressive infantile paralysis and premature death (Type I) to limited motor neuron loss and normal life expectancy (Type IV) (2, 3). SMA is caused by homozygous disruption in the survival motor neuron gene 1 (*SMN1*) (4, 5), whereas the disease severity is mainly influenced by the number of *SMN2* gene copies (6). A diagnosis of SMA has a profound impact on patients and their families (7, 8).

**Figure 1:**
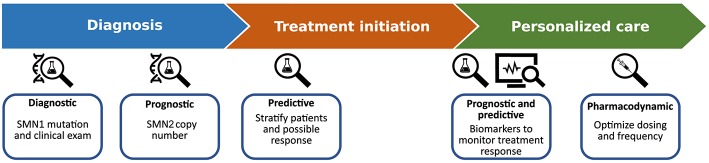
The utility of biomarkers in SMA treatment; current and future applications.

Recent advances in the demonstrated therapeutic efficacy of novel genetic and molecular therapies for SMA are fueling an unprecedented upsurge in clinical treatment (9). Phenotypic heterogeneity, that is inherent to this condition, may result in difficulties in providing early and accurate diagnosis, prognosis, assessment of disease activity and monitoring of treatment response. Within this dynamic setting the need for biomarkers to provide an objective measure is never more essential, to facilitate decision-making in clinical pathways for patients and guide therapeutic interventions in a tailored way (Figure 1).

Biomarkers may serve different purposes, but ideally share common key qualities (Table 1). These include stability in healthy individuals, with significantly different levels in disease cohorts to identify affected individuals. High degrees of sensitivity, specificity, precision, and reproducibility are also vital in an efficacious biomarker. In addition, biomarkers should reflect disease pathology, rather than disease epiphenomena and ideally be measured with ease, speed, and minimum expense in the target population.

**Table 1:** The classification of biomarkers.

Diagnostic	Facilitate detection of disease states when compared to healthy populations
Prognostic	Provide information on likely health outcomes, irrespective of treatment such as disease evolution or reoccurrence risk. Facilitate stratification of phenotypic severity
Predictive	Identify likely responders to treatment and patient populations
Pharmacodynamic	Confirm response to therapy Monitor therapeutic efficacy Surrogate endpoint in drug development and clinical trials (tightly linked to intended pharmacological targets and ensuing downstream processes)

**Figure 2:**
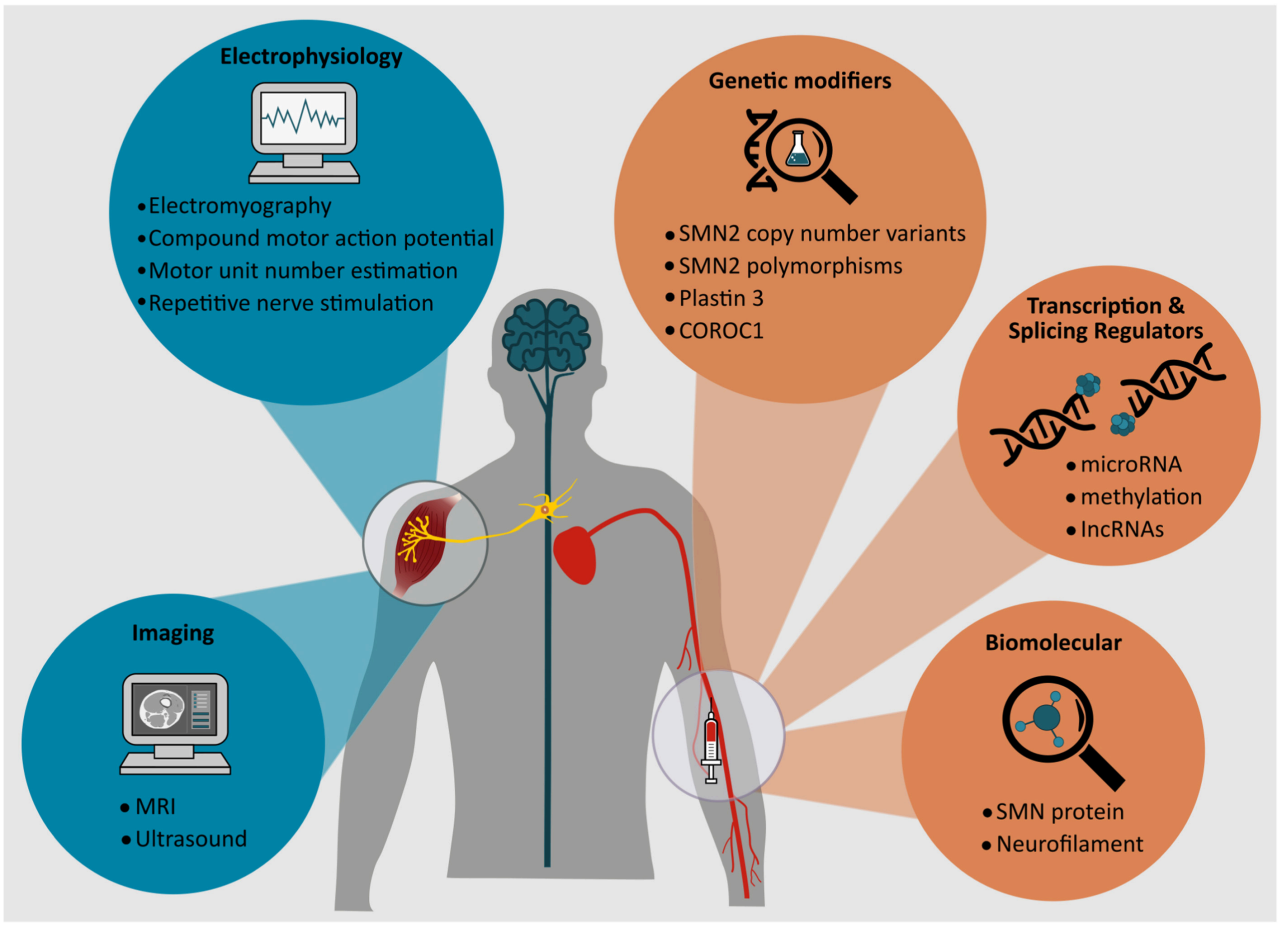
Potential electrophysiological, radiological, and circulating biomarkers for SMA.

This review will focus on the spectrum of candidate biomarkers in SMA, explore their role in facilitating our understanding of pathogenic mechanisms of disease and their clinical, pharmacological, and therapeutic utility. A variety of approaches are in early stages of discovery and development, encompassing biomolecular assays in serum and cerebrospinal fluid, as well as novel and conventional electrophysiological and neuroimaging assessments (Figure 2). A comprehensive understanding of factors that modify biological and pathogenic processes in SMA is therefore essential to realizing and curating efficacious biomarkers in this disorder.

## Circulating Biomarkers

Circulatory biomarkers have garnered significant attention to date, as tools for biomarker guided therapy in SMA. Their role spans a spectrum from prognostication, prediction of treatment response and monitoring the effects of therapeutic agents.

### Survival Motor Neuron Protein: The Cornerstone of SMA

SMA is caused by insufficient levels of the survival motor neuron (SMN) protein, due to biallelic *SMN1* deletion or mutation. The severity of SMA varies across a spectrum and is modified by the number of copies of the paralogous *SMN2* gene in humans, with the major difference conferred by a C to T nucleotide change in exon 7 (10, 11). This nucleotide change, though translationally silent, results in predominant skipping of exon 7 during *SMN2* pre-mRNA splicing, giving rise to a truncated transcript and protein (12, 13). Alternative splicing enables ~10% of *SMN2* transcripts to include exon 7 and produce a small amount of functional SMN (11, 14). SMN RNA and protein are ubiquitously expressed and have multiple roles in normal biological processes. These include general “housekeeping” and cell specific roles in ribonucleoprotein assembly, RNA metabolism (15), macromolecular trafficking, actin dynamics, and signal transduction (16). Alterations at any level of transcription, translation or splicing can lead to dysregulation of pathways involved in SMN protein production and potentially modify disease phenotype.

Therapeutic development has focused on augmenting SMN. The first approved drug for SMA (nusinersen), is an intrathecally delivered antisense oligonucleotide (ASO). Nusinersen was developed to alter the splicing of *SMN2* pre-mRNA by promoting inclusion of *SMN2* exon 7 by sequestering an inhibitory cis-element called Intronic Splicing Silencer N1 or ISS-N1, thus increasing concentrations of functional SMN protein (17, 18). Onasemnogene Abeparvovec (Zolgensma) is a one-time *SMN1* gene replacement therapy that may be administered intravenously or intrathecally (19). Several additional SMN induction therapies are currently in development, including systemic small molecules, such as risdiplam (20).

SMN protein levels are also dependent on degradation pathways. As such, the pathogenesis of SMA has been linked to mutations in the ubiquitin activating enzyme (UBA1) gene, encoding UBA1 that plays a crucial role in the ubiquitin proteasome system (UPS). Levels of SMN protein can be regulated by the UPS also making this a potential therapeutic target for SMA (21, 22). Previous studies have shown that pharmacological inhibition of the proteasome and UPS downstream targets can lead to phenotypic improvements in SMA mice (22–24). Findings from an animal study have identified an important role of SMN in the maintenance of ubiquitin homeostasis with decreased levels of UPS as a driving factor in SMA pathogenesis (24). There is a need for future studies to evaluate if different aspects of the UPS that are perturbed in SMA could act as potential drug targets independently or in combination with other SMN dependent strategies. With this clarification, ubiquitin pathways may in the future be proposed as putative mechanistic biomarkers of pharmacodynamic response to SMN enhancing therapies.

SMN protein is an obvious pharmacodynamic biomarker that can be measured from biological samples. This is aligned with therapeutic approaches designed to increase SMN levels. For example, preliminary data from clinical trials evaluating risdiplam, show >2-fold increase in *SMN2* protein levels in participants with SMA (20, 25). Similar results have been seen with salbutamol; a compound that increases *SMN2* full length transcript and SMN protein. In this study, a subjective improvement of motor function was noted in all patients with a statistically significant improvement in validated functional scores in a proportion of patients (26). This suggests target engagement and a potential method of tracking treatment response. Future studies are needed to determine the utility of SMN protein levels as a biomarker to guide dose optimization or frequency of the therapeutic regime.

There are limitations associated with SMN protein being used as a sole universal biomarker. For example, in one murine study, levels were unchanged in mice who were treated with ASO therapy, compared to an untreated cohort. Potentially, this could be attributed to utilizing blood assays to evaluate protein levels, when determining effects of CSF directed therapies (27). In a prospective analysis of SMN transcript and protein levels, as anticipated, plasma SMN protein was significantly lower in SMA samples compared to control. But, SMN protein levels did not correlate with *SMN2* copy number, disease severity or motor function (28). This lack of correlation has been attributed to modulation of SMN at the post-transcriptional level. Further studies have focused on assays of SMN protein levels in specific cell lines to circumvent variabilities of levels found in blood profiles. For example, spot analysis in peripheral blood nuclear cells, particularly CD33++ cells, has been shown to be a potential parameter of functional SMN protein levels (29). Additional validation studies will be necessary to demonstrate the efficacy of spot analysis.

### Neurofilaments

Neurofilaments (NF) are cytoplasmic proteins abundantly expressed in axons that have recently been recognized as promising diagnostic, prognostic, and monitoring biomarkers in a range of neurological disorders associated with axon loss (30–32). Initial discovery studies focused on modulation of *SMN2* encoded transcripts in children with SMA identifying NFs as potential biomarkers of disease activity and therapeutic response (33, 34). Across large nusinersen clinical trials with SMA types 1, 2 and presymptomatic infants (2 or 3 *SMN2* copies), plasma phosphorylated neurofilament heavy chain (pNF-H) differentiated SMA individuals from healthy controls. However, conclusions were limited by a small number of healthy pediatric age-matched controls (34, 35). Treatment initiation with nusinersen was associated with rapid decline followed by stabilization of pNF-H at levels close to those of healthy controls. pNF-H declined with advancing age in untreated patients, possibly due to reductions in motor neuron pool or disease activity. This raises uncertainty about its ability to demarcate whether the decline is due to physiological aging or a surrogate marker for treatment response. Serum neurofilament light chain (NfL) levels have also been evaluated in clinical trials investigating the safety and efficacy of branaplam, a small-molecule RNA splicing modulator. Preliminary results identified an inverse correlation between pre-treatment NfL levels and motor function scores in participants with SMA type I (36).

It is not yet clear which proteins released from motor neurons (NfL, Nf-H, or others) will be more sensitive in detecting the earliest stages of degeneration. These may have utility in the “presymptomatic” patient, helping guide decisions regarding when to start treatment. In addition, these may serve as pharmacodynamic markers, to verify suppression of continuing degeneration. Further evaluation of NFs across SMA populations and evaluation of potential correlations with efficacy outcomes is required.

## Genetic Modifiers

Genetic modifiers may enhance or suppress the effects of pathogenic mutations. Genetic modifiers also improve our mechanistic understanding of SMA and may identify novel targets for therapeutic intervention and future combination regimens. This knowledge serves to enhance our understanding of prognostic biomarkers in SMA, providing information on disease evolution and phenotypic severity.

### SMN2

The *SMN2* copy number is the most important modifier of clinical course in SMA, correlating inversely with age of symptom onset and severity (37–39), albeit with limitations in precision. Epidemiological studies demonstrate that more than 95% of individuals with ≤2 copies of *SMN2* have SMA type 1 with symptom onset in the first 6 months of life. Furthermore, <5% have SMA types 2 and 3 with symptom onset in early childhood (40–42). All major phenotypes of SMA are encompassed with ≤3 *SMN2* copies (40).

With the advent of newborn screening for SMA, *SMN2* copy number is emerging as a vital marker to guide the type and extent of intervention and stratify newborn patients to differing treatment arms. For example, there is consensus among experts that pre-symptomatic infants with ≤3 copies of *SMN2* should be promptly treated with disease modifying therapy, as they are predicted to have early onset forms (43). However, *SMN2* copy number has limitations, lacking precision as a biomarker of disease onset and prognosis. Theoretically, *SMN2* copy number acts in a dose-dependent manner to ameliorate the SMA phenotype. However, in observational studies, those with higher copy numbers do not always have a mild disease phenotype (44). This inherent limitation of *SMN2* as a prognostic biomarker, leads some experts to argue for disease-modifying treatment in all presymptomatic newborns with ≤4 *SMN2* copy numbers as identified through newborn screening programmes. Considerable overlap in copy number exists amongst phenotypic subgroups of patients with SMA (40). Furthermore, discordance in phenotype and response to therapy is noted in siblings with the same copy number, showing that there are other modifiers of disease at work in these individuals (45, 46). For example, sequence variations within the *SMN2* gene may positively modify phenotype, particularly the c.859G>C variant which increases inclusion of exon 7 and the amount of full length SMN transcript (47, 48). The latter is being used to stratify data analysis in current clinical trials (NCT03505099). Additional variants in *SMN2* introns 6 and 7 have been shown to alter the incorporation of exon 7 (49). Intron 6 variants (A-44G, A-549G, and C-1897T) have also been associated with milder SMA phenotypes in further studies (49, 50).

### Plastin 3

*Plastin 3* (*PLS3*) is a calcium dependent F-actin-binding protein (51). The latter forms an integral part of the axonal cytoskeleton and is thus crucial in a spectrum of cellular pathways, from axonal maturation to vesicular migration and endocytosis. PLS3 is a gender-specific, positive modifier, altering severity of SMA phenotype, in post-pubertal female patients only (52, 53). In murine models of SMA, PLS3 overexpression delays axonal pruning and rescues neuromuscular junction (NMJ) function (54). The molecular basis of PLS3 overexpression in unaffected individuals is unknown. Additionally, the degree to which PLS3 expression modifies SMA phenotype remains contentious, in part due to experiments in SMA mice showing no survival or electrophysiological benefit of PLS3 over-expression (55). A recent study analyzed effect of PLS3 on a panel of six potential biomarkers in mice (27). PLS3 overexpression neither influenced the SMN level nor the other experimental biomarkers, supporting the hypothesis that it acts as an independent protective modifier. Further studies are essential to translate these pre-clinical findings to the clinical sphere before the utility of PLS3 as a biomarker for SMA can be fully elucidated.

### Coronin 1C

Another phenotypic modifier is Coronin 1C that acts simultaneously on axons and muscles of the motor unit, by interacting with PLS3, in a calcium dependent manner to increase the amount of the F-actin (56). Additionally, PLS3 and Coronin 1C co-localize and work together in growth cones, axonal compartments and cell membranes of motor neurons (57). Both PLS3 and Coronin 1C rescue reduced vesicular pools in presynaptic terminals and restore NMJ function by facilitating endocytosis (55), leading to increased levels of F-actin. Consequently, in SMN depleted animal models where abnormal axonal branching and premature truncation are noted, this restoration of F-actin dynamics, ameliorate these structural anomalies and the SMA phenotype (56). Identification of this PLS3 interacting protein highlights the role of actin dynamics in pathomechanisms of SMA. Further research into protective modifiers such as PLS3 and Coronin 1C will provide improved prognostic genetic modifiers helping stratify disease severity and revealing potential targets for therapeutic intervention.

## Splicing Regulators as Modifiers of Phenotype

Aberrant splicing plays a significant role in SMA pathogenesis. Consequently, the development of biomarkers that accurately capture splicing events would greatly advance understanding of the control of SMN gene expression. Splicing regulators such as microRNA, epigenetic modifications and long non-coding RNA (lncRNA) may emerge as putative biomarkers.

### MicroRNA

MicroRNAs (miRNAs) regulate gene expression. Studies have shown their potential as non-invasive biomarkers in SMA (58). Differential expression of miRNAs (miR-9, miR-206, and miR-132) have been reported in spinal cord, skeletal muscle and serum from SMA and control mice, and in serum samples only from SMA and control patients. The SMA mice cohort presented with different severities (severe SMA-I and mild SMA-III) at different disease stages (presymptomatic, mid-symptomatic, and late stage). Serum miRNAs were altered prior to neuroanatomical changes in spinal cord and skeletal muscle at the presymptomatic stage. Especially, miR-132 was found to be most responsive to systemic ASO treatment in the severe mouse model (58). Thus, these experimental parameters may form the basis for an early diagnostic biomarker, particularly in the latent phase of disease, which can be used to track treatment response to *SMN2* enhancing therapies.

### Epigenetic Modifications: Methylation

Studies suggest that epigenetic effects such as *SMN2* methylation may regulate SMA disease phenotype by modulating its transcription (59–61). Genome wide methylation studies have determined differences in methylation patterns in certain genes, suggesting involvement of their proteins in pathogenesis of SMA (62). Discordant sibling pairs with identical SMN genotypes, suggest that epigenetic modification may control individual variations in the *SMN2* function (59).

### Long Non-coding RNAs

Recent advances have demonstrated that a significant portion of the genome is actively transcribed as non-coding RNA molecules. lncRNAs may be potential biomarkers of neuronal dysfunction due to their role in regulation of biological processes, and as “fine-tuners” of gene expression. SMA has long been considered not only a motor neuronal disease, but a process of anomalous neurodevelopment (63–66). The understanding of lncRNAs in SMA is still very limited. As proof of concept, one study has reported that targeting lncRNA to transcriptionally activate *SMN2*, in combination with *SMN2* splicing modification, ameliorates SMA. This demonstrates the promise of combinatorial ASO therapy in SMA (67). Future studies are needed to elucidate the prospect of lncRNAs as diagnostic and prognostic biomarkers and therapeutic targets.

## Electrophysiological Biomarkers

**Figure 3:**
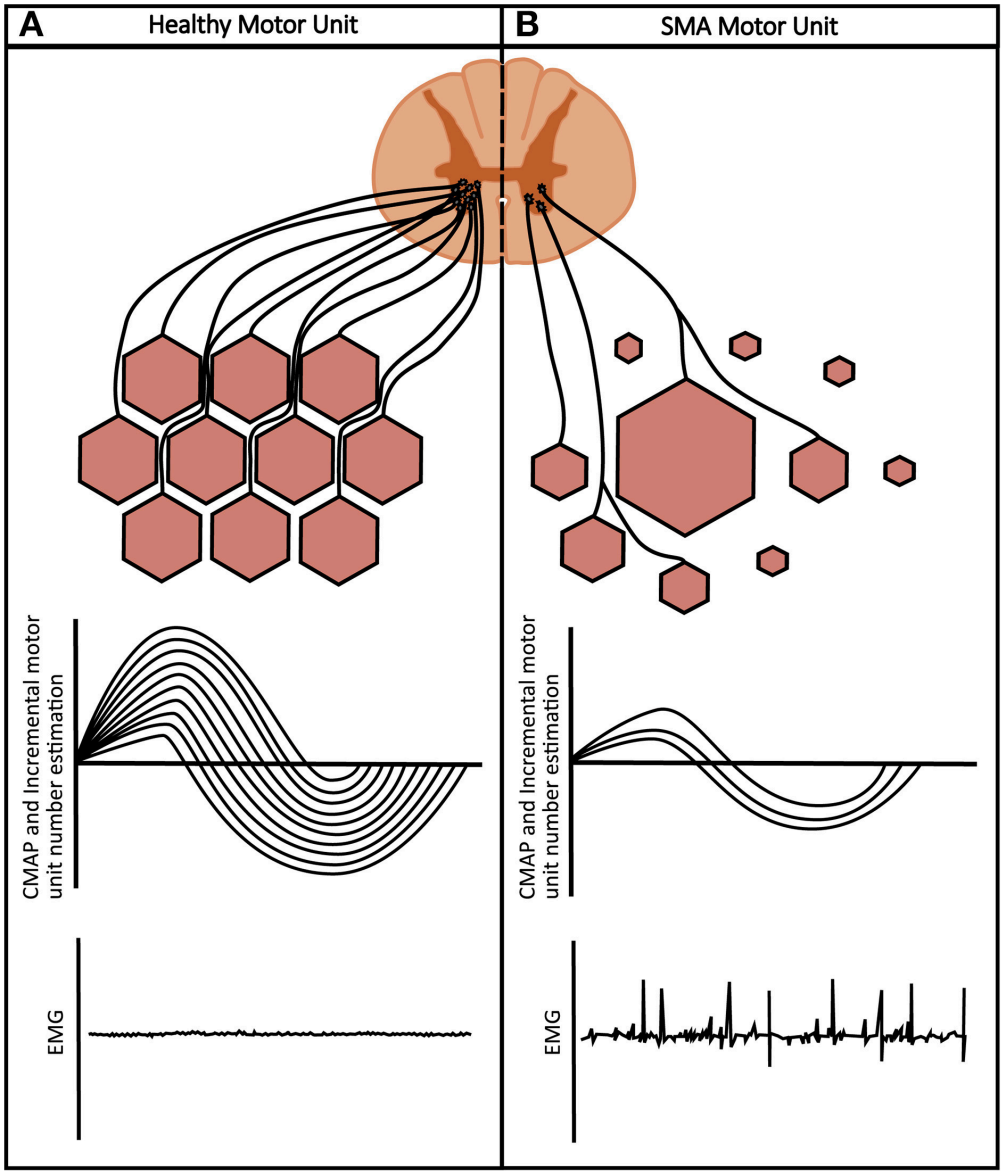
Healthy vs. SMA motor units: **(A)** healthy muscles are of uniform fiber size innervated by motor neurons. Neurophysiological measures show a high CMAP, full complement of incremental motor units and a silent EMG in relaxed muscle. **(B)** SMA muscle shows a mixture of small, denervated muscle fibers, and large hypertrophic units secondary to collateral reinnervation. Neurophysiological measures show a reduced CMAP, reduction in incremental motor unit number and fibrillations on EMG.

Serum biomarkers in SMA are in early phases of discovery and validation. Conversely, electrophysiological biomarkers have been studied, validated, and used globally in clinical trials in neuromuscular disease to assess the functional status of the motor unit pool *in vivo* (Figure 3) (68, 69).

### Compound Muscle Action Potential and Electromyography

A compound muscle action potential (CMAP) is obtained by providing a supramaximal stimulus to a motor nerve. The muscle response recorded is an indirect measure of the number of intact and available motor neurons, representing total functional status of the motor unit (70, 71). Electromyography (EMG) fibrillation potentials and positive sharp waves are spontaneous action potentials recorded from a relaxed muscle and generated by denervated muscle fibers (72). These parameters are proposed as easily collated potential biomarkers of disease in patients with SMA (73), measured with equipment used in the course of routine neurophysiology studies.

In murine models of presymptomatic SMA type 1, CMAP values are initially comparable to healthy controls. Subsequently a significant and rapid decline in CMAP correlates with a period of functional motor loss (disease onset) in affected mice (71). Similarly, a significant reduction in CMAP amplitude compared to healthy controls is noted in murine models of SMA type 2 and accompanied by needle EMG fibrillation potentials (71). When SMN restoration therapies in the form of ASOs are introduced, CMAP initially stabilizes, recovering gradually over time. Electrophysiological recovery correlates with functional recovery (71).

Clinical studies have also characterized CMAP as a surrogate marker of disease onset, status, and progression. A precipitous decline in CMAP is associated with sudden and significant functional decline in studies of prenatally defined patients (42). An ulnar CMAP ≤1.5 mV and evidence of denervation on EMG has thus been proposed as a threshold to consider disease onset in presymptomatic newborns, genetically confirmed with SMA. These electrophysiological parameters have also been incorporated into clinical trial design, to characterize the magnitude of motor neuron reserve at the time of treatment initiation in presymptomatic infants (18, 74).

The utility of the CMAP as a surrogate marker of disease status is replicated across all phenotypes of SMA. CMAP amplitude is found to discriminate between ambulant and non-ambulant patients in one cohort study (75). In patients with SMA significant reductions in CMAP correlate with reduced functional motor scores reproducibly over a number of studies (76, 77). Thus, CMAP measurement can potentially facilitate stratification of disease severity, as an adjunct to *SMN2* copy number. Patients with severe forms of SMA (≤2 copies of *SMN2*) show smaller and sometimes undetectable CMAP amplitudes, compared to those with milder genotypes (>2 copies of *SMN2*) (78).

The CMAP also holds potential as a biomarker of disease progression with patterns of decline dependent on phenotype. In a large natural history study of patients with SMA, overall CMAP declined significantly over the 5-year study period. In SMA type 1, this decline was predominantly in the first month of life, with relative stability thereafter. Patients with type II SMA have modest ongoing decline in CMAP amplitudes, whilst patients with SMA type III have relatively stable CMAPs throughout the study interval (42).

Longitudinal changes in CMAP have been examined as a secondary outcome measure and possible predictive biomarker of response to disease-modifying agents in clinical trials. The ENDEAR (phase 3 nusinersen) reported that motor milestone response occurred in 41% of patients, and with CMAP response in 36% treated infants. CMAP could therefore be adjunctive to longitudinal changes in functional motor scores to assess treatment response to therapeutic intervention (18). In children with later onset SMA, treatment with nusinersen over several years produced improvements in motor function, concomitant with stable CMAP readings (79). Further detailed neurophysiological assessments may provide a deeper understanding of treatment response.

The CMAP is also being explored as a putative prognostic biomarker. Natural history studies have shown that maximal baseline CMAP has a relative prognostic value in determining functional outcome in untreated patients with SMA (42). This putative prognostic biomarker may determine the severity of pre-existing denervation and be useful in setting realistic goals for therapeutic intervention (42). However, baseline CMAP has limitations in predicting other important clinical outcomes, with studies determining that it does not correlate with risk and age of death or use of permanent ventilation (78).

Standardized operating protocols are essential to ensure the CMAP is a feasible, valid, reliable, and reproducible outcome measure. Limiting factors such as background noise, inconsistent electrode placement and contact, may produce large errors, particularly in the small CMAP measurements evident in late disease stages (75, 79, 80). Patient tolerance and cooperation (to avoid movement and EMG artifact) is also critical to success. Specificity of these measures is also low. Localizing areas of pathology within the motor unit and consequently pinpointing targets of therapeutic intervention, is impossible with such qualitative markers. Dysfunction at any point along the motor unit can contribute to changes in CMAP amplitudes and development of fibrillation potentials (81). Additionally, CMAP amplitudes may be preserved despite loss of motor neurons due to compensatory changes (collateral reinnervation). This is especially noted in earlier stages prior to loss of significant proportions of motor units (68, 82). Therefore, other measures are essential to complement CMAP to determine the motor neuron input to muscles and show progression of disease through falling numbers of motor units (42).

### Motor Unit Number Estimation and Single Motor Unit Potentials

Motor unit number estimation (MUNE) provides an assessment of axon number and the capacity for reinnervation represented by size of the average single motor unit potential (SMUP). These parameters are linked closely to pathophysiology in SMA (42, 68, 83–85). Furthermore, in comparison to clinical measures of strength and function in SMA, MUNE as an objective measure is not limited by effort, fatigue, contractures, or developmental stage. Multiple ways of estimating MUNE exist. An incremental MUNE technique uses increasing strengths of submaximal stimuli to recruit motor units into the firing pool to determine the average SMUP (86). Traditional MUNE methods are derived from the basis that maximum CMAP is a composite measure of the number of functional motor units and the average amplitude made by a single motor unit (87).

In rodent models of SMA, MUNE values are significantly reduced. Declines in MUNE correlate directly with emergence of the motor phenotype (71). Administration of ASOs correlate with improvement in phenotype and MUNE values, reflecting preservation of the threatened motor unit pool (71, 88). This effect is enhanced at points distant to the time of administration, possibly depicting ongoing effects of intervention (71).

MUNE is proposed as a diagnostic marker of disease onset, as values in symptomatic children with SMA remain significantly below that of healthy infants (42). In small numbers of pre-symptomatic neonates and infants, changes in MUNE value were highly sensitive to early deterioration in the motor unit pool and a precursor to manifestation of clinical symptoms (18, 42). MUNE may also be helpful in the broad stratification of SMA phenotype, with lower values generally noted in the more severe forms (SMA type I), compared to milder phenotypes (SMA type III) (89). However, considerable overlap exists in MUNE values between SMA phenotypes (42), and thus its use for predicting disease severity may have limitations.

In some studies, MUNE values appear to be highly dependent on and inversely related to disease duration in untreated patients (42), however stability in the chronic phase may be apparent for many individuals. Other studies demonstrate increases in MUNE in untreated subjects while CMAPs remain stable longitudinally. This leads the suggestion that new motor unit development may occur as a compensatory response to motor unit loss (73). Alternatively, these findings may be linked to a normal developmental, maturational process, causing collateral innervation with polysynaptic connections amongst motor units, which are pruned, becoming monosynaptic as motor units physiologically mature (90). In treated cohorts, MUNE has been assessed over the longer term in phase 1/2 studies of nusinersen, demonstrating relative stability of values in children with SMA type II, compared with longitudinal declines in SMA type III, despite improvements in functional scores in the latter group (79). These findings may be explained by the focus of MUNE on distal muscles such that it may not reflect the proximal effects of novel therapies. For example, distal reinnervation may be difficult to achieve, especially where a high demand for SMN protein exists to maintain and increase motor unit capacity, in the growing phase of a child with later onset forms of SMA (79). Because of these differing findings, MUNE may not be helpful when used in isolation as a surrogate biomarker of disease progression and response to treatment. A more personalized approach, accounting for disease duration and severity may enable MUNE to be more judiciously used as a precision biomarker. Additional studies are vital to further understand MUNE values within the dynamic setting of pathological and normal developmental pathways of neuronal denervation/reinnervation, and to establish lower-limit normative values (91).

Variability in MUNE may be influenced by the operator's expertise, such that standardized procedures and training are critical. Different MUNE techniques have been developed, varying in procedures and the manner that SMUPs are calculated. The methodology of deriving MUNE may thus change the sensitivity, specificity, and reproducibility of this biomarker. Early MUNE methods were derived from the basis that the maximum CMAP is a composite measure of the number of functional motor units and the average amplitude made by a single motor unit as it is “recruited” into firing by submaximal stimulation (86). Average SMUP size is traditionally derived by increasing stimulus size to recruit increasing numbers of individual motor units into the firing pool and averaging the amplitude of these units (incremental method). In SMA, this method of deriving MUNE correlates well with functional motor scores, particularly sensitive to severity of weakness. Average SMUP size shows negative correlation to motor score, perhaps denoting a process of reinnervation with increasing severity of weakness. These findings confirm the incremental method's potential utility in showing motor neuron loss and tracking decompensation/compensation changes through SMUP (85). Longitudinal studies are awaited to confirm these results.

There are inherent limitations of incremental methods. These affect the accuracy of motor unit estimation (92). Other methodologies such as multi-point method samples the first all-or-none SMUP response to a low-level stimulus, over repeated points along the motor nerve to collate an average sample of SMUPs (93). An adapted system, combining incremental stimulus and multiple point analysis is increasingly used to improve estimate accuracy. Novel, computerized methods of MUNE are coming to the fore including the MScan fit and MUNIX methods, to improve analysis accuracy and time, and circumvent biases incurred by operator input, which are intrinsic weaknesses noted in traditional methods (94, 95). Their clinical role is yet to be elucidated in terms of sensitivity, specificity, and reproducibility in healthy controls and for individuals with SMA.

## Neuroimaging Biomarkers

### Electrical Impedance Myography

Electrical impedance myography (EIM) summates surface muscle action potentials derived from direct application of a low-intensity, high frequency stimulus to muscle. The method involves quantitative measurement of changing parameters in the muscle's extra and intracellular fluid resistance, and cell membrane reactance (as a measure of its capacitance), to a sinusoidal current, applied at varying frequencies (96). This measure reflects the changing intrinsic properties of the muscle (97), denoting tissue quality, including changes secondary to fibrosis, denervation, and edema (98). The adult onset disease amyotrophic lateral sclerosis (ALS) has acted as a model for biomarker exploration in disease of upper and lower motor neurons. Findings in this condition may therefore be extrapolated to other motor neuron pathologies such as SMA. In studies of disease progression in ALS, this method correlates well with traditional MUNE methods, and validated functional motor scores. It is sensitive to subtle disease progression (99), easy to perform with little technical training required, and allows proximal muscles to be sampled. Use of EIM was purported to reduce sample size significantly in one multi-center trial (100). Its use in tracking therapeutic interventions secondary to muscle-specific therapies such as myostatin inhibitors is also theoretically possible. However, its utility has significant limitations. For instance, its specificity as a diagnostic identifier of ALS, when compared to ALS-mimics, is low (100). Furthermore, in a small clinical trial of EIM in children with SMA, this parameter remained static over the study period, when compared to healthy controls where EIM showed non-mass dependant muscle maturation (101). It is difficult to know if this reflects lack of muscle maturation, absence of decline in muscle fibers, or a rate of decline that counteracts maturation potential in disease cohorts (101).

### MRI and Ultrasound

Magnetic resonance imaging (MRI) of skeletal muscle has been used in small numbers of pre-clinical and clinical studies to provide alternative markers of disease. Muscle composition changes with disease duration in SMA (102). Changes in parameters such as muscle fat fraction correlate well with validated functional motor scores in non-SMA motor neuropathies and are highly sensitive to disease progression (103). Ultrasound offers a different modality for assessment of muscle composition in patients with motor neuropathies. Muscle thickness and echo intensity have been reviewed to a limited extent in ALS, where one study showed use in prognosticating survival (104). Longitudinal studies have noted a reduction in hand muscle cross-sectional area in ALS disease cohorts (105), but these changes correlate poorly with functional abilities and may therefore not be a sensitive representative of disease progress (106).

Significant work needs to be done before MRI or US can be purported as a suitable method for producing viable biomarkers in SMA. Firstly, it is necessary to trace changes in muscle composition and architecture in healthy controls over time, to establish normal age-dependant baselines. Furthermore, standardized protocols encompassing type and number of muscles assessed, techniques for evaluation, and comparability of equipment used is pivotal before the feasibility and reproducibility of these biomarkers can be elucidated.

## Exploratory Biomarkers of the Neuromuscular Junction

### Repetitive Nerve Stimulation

Whilst SMA is considered a primary neurogenic process, it is increasingly recognized that it is associated with concomitant dysfunction at the level of the NMJ, secondary to lack of the SMN protein required for normal NMJ development and maturation (107–109). Abnormalities in NMJ impulse transmission may be responsible for significant degrees of fatigability commonly reported by affected individuals, and clinically observed when using validated measures of endurance such as the 6-min walk test (110–112). Therefore, an electrophysiological biomarker such as repetitive nerve stimulation (RNS) to determine the presence and extent of NMJ dysfunction (113), may facilitate a different modality of looking at pathophysiology and response to novel therapies. In a recent study of SMA cohorts, a pathological decrement on low frequency RNS was noted in 49% of patients with SMA but not in healthy controls or patients with other motor neuron diseases (108). This decrement was independent of SMA subgroup, clinical score, and disease duration (108). Therapies directed toward augmentation of NMJ impulse transmission provide a novel target, with pyridostigmine currently being assessed in a phase II clinical trial (NCT02941328) (114). Thus, RNS may be helpful as a biomarker of treatment response in therapies that augment NMJ function.

## Conclusion

Within the changing SMA therapeutic landscape there are many current clinical uncertainties, including determination of optimal timings, regimen, mode, and response to treatment. The target treatment population and prognostication of meaningful endpoints for patients also require definition. A repertoire of validated biomarkers is essential. This will enable a comprehensive evaluation of SMA and facilitate clinical decision-making across diagnosis, prognosis, pharmacotherapy, and support future research advances. While numerous genetic, epigenetic, proteomic, electrophysiological, and imaging biomarkers have been suggested, future studies are needed to determine reproducibility across the SMA population. Thus far, *SMN2* appears to be a highly valuable prognostic biomarker in SMA. Its utility includes stratification of patients for treatment in research and clinical spheres. Better understanding of other genetic and epigenetic modifiers would facilitate an individualized approach to prognostication. More recently, neurofilament has been proposed as a pharmacodynamic biomarker of disease response to treatment and is being investigated across the phenotypic spectrum of SMA. Neurophysiological parameters of motor unit function include CMAP and MUNE assessments. These have been used primarily as surrogate markers of disease onset and to track disease progression in treated and untreated cohorts. Emerging data suggests that they may also have a potential role in disease prognostication and help predict treatment responders. Nevertheless, there may not be a “one size fits for all” biomarker. Different biomarkers may be required to assess specific questions related to disease progression, treatment efficacy, safety, and prognostic endpoints. Suitable biomarkers may change depending on the therapeutic target of medical intervention, as increasing number of agents come to the fore and compete for use in patients with SMA. As a collective, the ultimate aim of these biomarkers is to enable a personalized approach to management, facilitating a smooth and optimal pathway for the patient through their clinical journey.

## Author Contributions

DK, AD'S, and MF planned the manuscript. DK and AD'S executed and prepared the first and subsequent drafts of the manuscript. CL, MR, and MF contributed to manuscript revision. All authors read and approved the submitted version.

### Disclosure

MF and MR had received honoraria for scientific advisory boards from Biogen, Roche and AveXis. AD'S received funding from the Sydney Children's Hospital Foundation Researcher Grant (RG182501).

### Conflict of Interest Statement

The authors declare that the research was conducted in the absence of any commercial or financial relationships that could be construed as a potential conflict of interest.
